# Time-resolved small RNA transcriptomics of the ichthyosporean *Sphaeroforma arctica*

**DOI:** 10.12688/f1000research.133935.1

**Published:** 2023-05-24

**Authors:** Andrej Ondracka, Omaya Dudin, Jon Bråte

**Affiliations:** 1 Institut de Biologia Evolutiva (CSIC-Universitat Pompeu Fabra), Barcelona, Spain; 2 Swiss Institute for Experimental Cancer Research, School of Life Sciences, Swiss Federal Institute of Technology (EPFL), Lausanne, 1015, Switzerland; 3 Department of Biosciences, University of Oslo, Oslo, 0316, Norway; 4 Department of Virology, Norwegian Institute of Public Health, Oslo, 0456, Norway

**Keywords:** Ichthyosporea, Sphaeroforma arctica, small RNA, microRNA, miRNA, gene regulation, cell differentiation, origin of animals

## Abstract

Ichthyosporea, a clade of holozoans, represent a clade closely related to animals, and thus hold a key phylogenetic position for understanding the origin of animals. We have previously discovered that an ichthyosporean, *Sphaeroforma arctica,* contains microRNAs (miRNAs) as well as the miRNA processing machinery. This was the first discovery of miRNAs among the closest single-celled relatives of animals and raised intriguing questions about the roles of regulatory small RNAs in cell development and differentiation in unicellular eukaryotes. Like many ichthyosporeans, *S. arctica* also undergoes a transient multicellular developmental life cycle. As miRNAs are, among other roles, key regulators of gene expression during development in animals, we wanted to investigate the dynamics of miRNAs during the developmental cycle in *S. arctica.* Here we have therefore collected a comprehensive time-resolved small RNA transcriptome linked to specific life stages with a substantially higher sequencing depth than before, which can enable further discovery of functionally relevant small RNAs. The data consists of Illumina-sequenced small RNA libraries from two independent biological replicates of the entire life cycle of *S. arctica* with high temporal resolution. The dataset is directly linked and comes from the same samples as a previously published mRNA-seq dataset, thus enabling direct cross-functional analyses.

## Introduction

Ichthyosporeans hold a key position in the evolutionary tree for understanding the origin of animals and animal multicellularity. Ichthyosporea is a clade of holozoans, of which many characterized representatives undergo multinucleate (coenocytic) life cycles and exhibit a transient multicellular stage during cellularization of the coenocytes. ^1^^,^^2^ Among ichthyosporeans, the life cycle of *Sphaeroforma arctica* has been best characterized. Multiple nuclear division cycles in a single cell occur with highly regular timing, forming multinuclear coenocytes, ^3^ which is followed by actomyosin-dependent cellularization. ^4^ The whole life cycle has been characterized through mRNA transcriptomics, showing dynamic transcriptional regulation during the life cycle, including transcriptional regulation of the putative key regulators of cellularization. ^4^

microRNAs (miRNAs) are short RNA molecules that, among many roles, regulate the activity of genes important for multicellular development in both animals and plants (e.g., Refs. 5, 6). Although miRNAs have been reported from other eukaryote groups spanning the tree of life, such as brown and green algae (e.g., Refs. 7, 8) Amoebozoa (e.g., Ref. 9) excavates (e.g., Ref. 10) and unicellular Holozoa, ^11^ their presence and function in many cases remain controversial. ^12^ Nevertheless, these discoveries raise the intriguing question of whether small regulatory RNAs also play a role during development in unicellular and facultatively multicellular organisms. To understand whether miRNAs play a role in its development, we collected a high-quality small RNA dataset in *S. arctica* at both high depth and high temporal resolution throughout its entire developmental cycle.

## Methods

The purpose of this study was to generate a dataset in order to (1) investigate the temporal dynamics of miRNA expression in *S. arctica* and to provide potential functional insights into the miRNAs, (2) discover novel miRNA genes in *S. arctica*, and (3) discover other potentially functionally relevant small RNAs, such as piRNAs (e.g., Refs. 13, 14).

We have acquired high throughput sequencing datasets of the fraction of RNA molecules smaller than approximately 200 nucleotides (small RNAs). We have isolated and sequenced the small RNA content from synchronized cultures every six hours over a period of 72 hours, spanning the entire cell cycle. The sequencing was done in two biological replicates. The experiment was performed in parallel with the previously published mRNA transcriptome dataset ^4^ and the small RNA libraries were prepared from the same total RNA samples; thus, the present dataset can be analyzed simultaneously with the mRNA data.

### Experimental design and culturing conditions and RNA extraction

The cultures were prepared according to the protocol originally described in Ref. 4. In detail, *S. arctica* cells were cultured in Marine Broth media (Marine Broth, Difco BD, NJ, USA; 37.4 g/L) in sterile culture flasks at 12°C in dark conditions. The cultures were grown to a stationary phase, which has been shown to synchronize the coenocytic cycles (see Ref. 3). At the start of the experiment, the saturated cultures were diluted 1:300 into fresh marine broth media. Aliquots were collected every six hours for 72 hours, spanning an entire coenocytic life cycle. RNA was extracted using the miRNeasy Mini Kit (QIAGEN, Venlo, Netherlands) from approximately 50 mL of culture for each culture aliquot, and RNA integrity was evaluated using a Bioanalyzer 2100 (Agilent, CA, US).

### Library preparation and sequencing details

The small RNA libraries were prepared using the NEBNext Small RNA Library Prep Set for Illumina (New England Biolabs, MA, US). A total of 50 bp single-end reads were obtained by sequencing the libraries on the Illumina HiSeq 2500 platform with the v4 chemistry and high output mode. Library preparation and sequencing was carried out by the CRG Genomics core unit, Barcelona, Spain. The data presented here is not processed in any way. The sequencing libraries contained on average 22.7 million reads (SD = 5.3 mill reads). Together, this data represents a more than 40-fold higher sequencing depth than the previous study, ^11^ where small RNA reads were obtained from only two samples.

### Limitations

The small RNA extractions were sequenced as is, without any external, or spiked-in, controls.

## Data Availability

European Nucleotide Archive: *Sphaeroforma arctica* small RNAs. Accession number PRJEB55646; https://identifiers.org/ena.embl:PRJEB55646 Table 1 details the individual files under accession number PRJEB55646. Data are available under the terms of the Creative Commons Zero “No rights reserved” data waiver (CC0 1.0 Public domain dedication).

## References

[1] Mendoza L Taylor JW Ajello L : The class mesomycetozoea: a heterogeneous group of microorganisms at the animal-fungal boundary. *Annu. Rev. Microbiol.* 2002;56:315–344. 10.1146/annurev.micro.56.012302.160950 12142489

[2] Jøstensen J-P Sperstad S Johansen S : Molecular-phylogenetic, structural and biochemical features of a cold-adapted, marine ichthyosporean near the animal-fungal divergence, described from in vitro cultures. *Eur. J. Protistol.* 2002;38:93–104. 10.1078/0932-4739-00855

[3] Ondracka A Dudin O Ruiz-Trillo I : Decoupling of Nuclear Division Cycles and Cell Size during the Coenocytic Growth of the Ichthyosporean Sphaeroforma arctica. *Curr. Biol.* 2018;28:1964–1969.e2. 10.1016/j.cub.2018.04.074 29887314 PMC6013282

[4] Dudin O Ondracka A Grau-Bové X : A unicellular relative of animals generates a layer of polarized cells by actomyosin-dependent cellularization. *elife.* 2019;8:e49801. 10.7554/eLife.49801 31647412 PMC6855841

[5] Bartel DP : Metazoan MicroRNAs. *Cell.* 2018;173:20–51. 10.1016/j.cell.2018.03.006 29570994 PMC6091663

[6] Moran Y Agron M Praher D : The evolutionary origin of plant and animal microRNAs. *Nat. Ecol. Evol.* 2017;1:0027. 10.1038/s41559-016-0027 28529980 PMC5435108

[7] Cock JM Liu F Duan D : Rapid Evolution of microRNA Loci in the Brown Algae. *Genome Biol. Evol.* 2017;9:740–749. 10.1093/gbe/evx038 28338896 PMC5381526

[8] Molnár A Schwach F Studholme D : miRNAs control gene expression in the single-cell alga Chlamydomonas reinhardtii. *Nature.* 2007;447:1126–1129. 10.1038/nature05903 17538623

[9] Avesson L Reimegård J Wagner EG : MicroRNAs in Amoebozoa: deep sequencing of the small RNA population in the social amoeba Dictyostelium discoideum reveals developmentally regulated microRNAs. *RNA.* 2012;18:1771–1782. 10.1261/rna.033175.112 22875808 PMC3446702

[10] Huang PJ Lin WC Chen SC : Identification of putative miRNAs from the deep-branching unicellular flagellates. *Genomics.* 2012;99:101–107. 10.1016/j.ygeno.2011.11.002 22120185

[11] Bråte J Neumann RS Fromm B : Unicellular Origin of the Animal MicroRNA Machinery. *Curr. Biol.* 2018;28:3288–3295.e5. 10.1016/j.cub.2018.08.018 30318349 PMC6206976

[12] Tarver JE Donoghue PCJ Peterson KJ : Do miRNAs have a deep evolutionary history? *BioEssays.* 2012;34:857–866. 10.1002/bies.201200055 22847169

[13] Ozata DM Gainetdinov I Zoch A : PIWI-interacting RNAs: small RNAs with big functions. *Nat. Rev. Genet.* 2019;20:89–108. 10.1038/s41576-018-0073-3 30446728

[14] Grimson A Srivastava M Fahey B : Early origins and evolution of microRNAs and Piwi-interacting RNAs in animals. *Nature.* 2008;455:1193–1197. 10.1038/nature07415 18830242 PMC3837422

