# Supplementary material for: Time-resolved small RNA transcriptomics of the ichthyosporean Sphaeroforma arctica
Source: F1000Res. 2023 May 24;12:542. [Version 1] doi: 10.12688/f1000research.133935.1 (PMC11109566; doi:10.12688/f1000research.133935.1)
Supplement: Peer review history

# Reviewer report: Reviewer response for version 1

**DOI:** 10.5256/f1000research.146953.r248045

**Published:** 2024-05-11

**Authors:** Arkadiy Garber

**Affiliations:** 1 Arizona State University, Tempe, Arizona, USA

In this data report, Ondracka and Dudin present a number of small RNA libraries corresponding to different life stages of ichthyosporean Sphaeroforma arctica. I believe that this data will be very useful to the research community. My only recommendation to the methods would be to more explicitly state how S. arctica cells were processed for RNA extraction. For example, how were the cells lysed? Was there any centrifugation to pellet the cells or cell debris? Was there any rRNA depletion done during library prep?

Are sufficient details of methods and materials provided to allow replication by others?

Partly

Is the rationale for creating the dataset(s) clearly described?

Yes

Are the datasets clearly presented in a useable and accessible format?

Yes

Are the protocols appropriate and is the work technically sound?

Yes

Reviewer Expertise:

My area of expertise is in microbiology, bioinformatics, and evolutionary biology. I also have a background in molecular biology (including DNA and RNA).

I confirm that I have read this submission and believe that I have an appropriate level of expertise to confirm that it is of an acceptable scientific standard.

# Reviewer report: Reviewer response for version 1

**DOI:** 10.5256/f1000research.146953.r252697

**Published:** 2024-04-01

**Authors:** Rafał Milanowski

**Affiliations:** 1 University of Warsaw, Warsaw, Poland

Ichthyosporea is a group of Opisthokonta that plays a crucial role in discussions about the origins of animals and animal multicellularity. The Data Note article by Ondracka et al. describes the previously unpublished small RNA transcriptome associated with specific life stages of the ichthyosporean species Sphaeroforma arctica. This dataset holds the potential to facilitate further discovery of functionally relevant small RNAs in this species.

 The methodological section of the manuscript provides detailed procedures for culturing, RNA extraction, library preparation, and sequencing, thereby enabling the replication of all experiments. Data files are well-organized, summarized in a table, and deposited in the RNA database under a unique accession number. The references are thoughtfully selected, offering opportunities for deeper exploration of the topic.

Are sufficient details of methods and materials provided to allow replication by others?

Yes

Is the rationale for creating the dataset(s) clearly described?

Yes

Are the datasets clearly presented in a useable and accessible format?

Yes

Are the protocols appropriate and is the work technically sound?

Yes

Reviewer Expertise:

Phylogeny, evolution and genomics of protists

I confirm that I have read this submission and believe that I have an appropriate level of expertise to confirm that it is of an acceptable scientific standard.
